# Site‐directed mutagenesis improves the practical application of L‐glutamic acid decarboxylase in *Escherichia coli*

**DOI:** 10.1002/elsc.202200064

**Published:** 2023-03-09

**Authors:** Liu Fengmin, Zhang Heng, Zhang Xiangjun, Wei Xiaobo, Liu Huiyan, Fang Haitian

**Affiliations:** ^1^ School of Food and Wine Ningxia Key Laboratory for Food Microbial‐Applications Technology and Safety Control Ningxia University Yinchuan China

**Keywords:** γ‐aminobutyric acid, glutamate decarboxylase, site‐directed mutation, whole cell transformation

## Abstract

γ‐Aminobutyric acid (GABA) is a kind of non‐proteinogenic amino acid which is highly soluble in water and widely used in the food and pharmaceutical industries. Enzymatic conversion is an efficient method to produce GABA, whereby glutamic acid decarboxylase (GAD) is the key enzyme that catalyzes the process. The activity of wild‐type GAD is usually limited by temperature, pH or biotin concentration, and hence directional modification is applied to improve its catalytic properties and practical application. GABA was produced using whole cell transformation of the recombinant strains *Escherichia coli* BL21(DE3)‐Gad B, *E. coli* BL21(DE3)‐Gad B‐T62S and *E. coli* BL21(DE3)‐Gad B‐Q309A. The corresponding GABA concentrations in the fermentation broth were 219.09, 238.42, and 276.66 g/L, and the transformation rates were 78.02%, 85.04%, and 98.58%, respectively. The results showed that Gad B‐T62S and Gad B‐Q309A are two effective mutation sites. These findings may contribute to ideas for constructing potent recombinant strains for GABA production.

***Practical Application***: Enzymatic properties of the GAD from *Escherichia coli* and GAD site‐specific mutants were examined by analyzing their conserved sequences, substrate contacts, contact between GAD amino acid residues and mutation energy (ΔΔG) of the GAD mutants. The enzyme activity and stability of Gad B‐T62S and Gad B‐Q309A mutants were improved compared to Gad B. The kinetic parameters K_m_ and V_max_ of Gad B, Gad B‐T62S, and Gad B‐Q309A mutants were 11.3 ± 2.1 mM and 32.1 ± 2.4 U/mg, 7.3 ± 2.5 mM and 76.1 ± 3.1 U/mg, and 7.2 ± 3.8 mM and 87.3 ± 1.1 U/mg, respectively. GABA was produced using whole cell transformation of the recombinant strains *E. coli* BL21(DE3)‐Gad B, *E. coli* BL21(DE3)‐Gad B‐T62S, and *E. coli* BL21(DE3)‐Gad B‐Q309A. The corresponding GABA concentrations in the fermentation broth were 219.09, 238.42, and 276.66 g/L, and the transformation rates were 78.02%, 85.04%, and 98.58%, respectively.

**Abbreviations**
DE3: E. coli BL21
E. coli: Escherichia coli
GABA: γ‐Aminobutyric acid
GAD: glutamic acid decarboxylase
IPTG: isopropyl thiogalactoside
PLP: pyridoxal phosphate

## 1 INTRODUCTION

γ‐Aminobutyric acid (GABA) is a four‐carbon non‐proteinogenic amino acid that is highly soluble in water and widely distributed in animals and plants. GABA plays an important role in improving the physiological function of the body and is often used as a drug and food addition. In the pharmaceutical industry, GABA has an anticonvulsant function [1] and is used to treat epilepsy [2], anxiety [3], and in adjuvant treatment of Parkinson's disease [4]. As a food addition, GABA is often added to beverages, alcohol, and milk to prevent neurological diseases [5]. In addition to its application in preventing plant diseases and promoting plant photosynthesis [6], GABA has also attracted increasing attention as a postharvest treatment to inhibit the browning of mushrooms such as *Agaricus bisporus* [7].

GABA can be synthesized by chemical, plant enrichment, and biological methods [8, 9]. Chemical production of GABA has poor safety and high energy consumption, and the acquisition of raw materials and waste disposal are relatively cumbersome [10]. The plant enrichment production method of GABA has low efficiency and the separation is difficult, which will result in a reduced GABA yield [11]. As a result, the biological synthesis of GABA has attracted a lot of attention due to its advantages of simple raw material requirements, an easy process and environmental friendliness [12]. The biological methods can be divided into fermentation and transformation methods [13]. Pham et al. and Vo et al. [14, 15], In order to improve the efficiency of the GABA pathway a synthetic protein scaffold was introduced in *E. coli* and *P. horikoshii* glutamic acid decarboxylase was overexpressed so that the recombinant strains successfully produced GABA from glucose. Chae et al. [16], successfully constructed an engineered bacterium with glutamate decarboxylase (GAD) activity by transforming the GAD of *E. coli* K‐12 into *E. coli* BL21(DE3) that used 300 mM sodium glutamate as the substrate for whole cell transformation to produce up to 102 mM of GABA. Fan et al. [17], performed site‐directed saturation mutagenesis of the N‐terminal residues of GadB from *E. coli* to improve its thermal stability. A triple mutant (M6, Gln5Ile/V al6Asp/Thr7Gln) showed higher thermal stability. GAD is the key enzyme catalyzing GABA production from L‐glutamate [18], and its activity directly determines the amount of GABA obtained by the transformation method. Generally, GAD from wild strains has low activity and poor stability, which limits its application [19, 20]. Nevertheless, the application of site‐directed mutagenesis in enzyme transformation provides a new potential for the increased production of GABA. On the basis of multiple sequence alignment, the enzyme activity center, protein surface amino acids, protein model, and other factors, site‐directed mutation of relevant amino acid residues of *Bacillus megaterium* GAD was used for cell transformation by Cheng HJ [21] and the studies managed to produce 347.9 g/L GABA from 500 g/L L‐glutamate.

**FIGURE 1:**
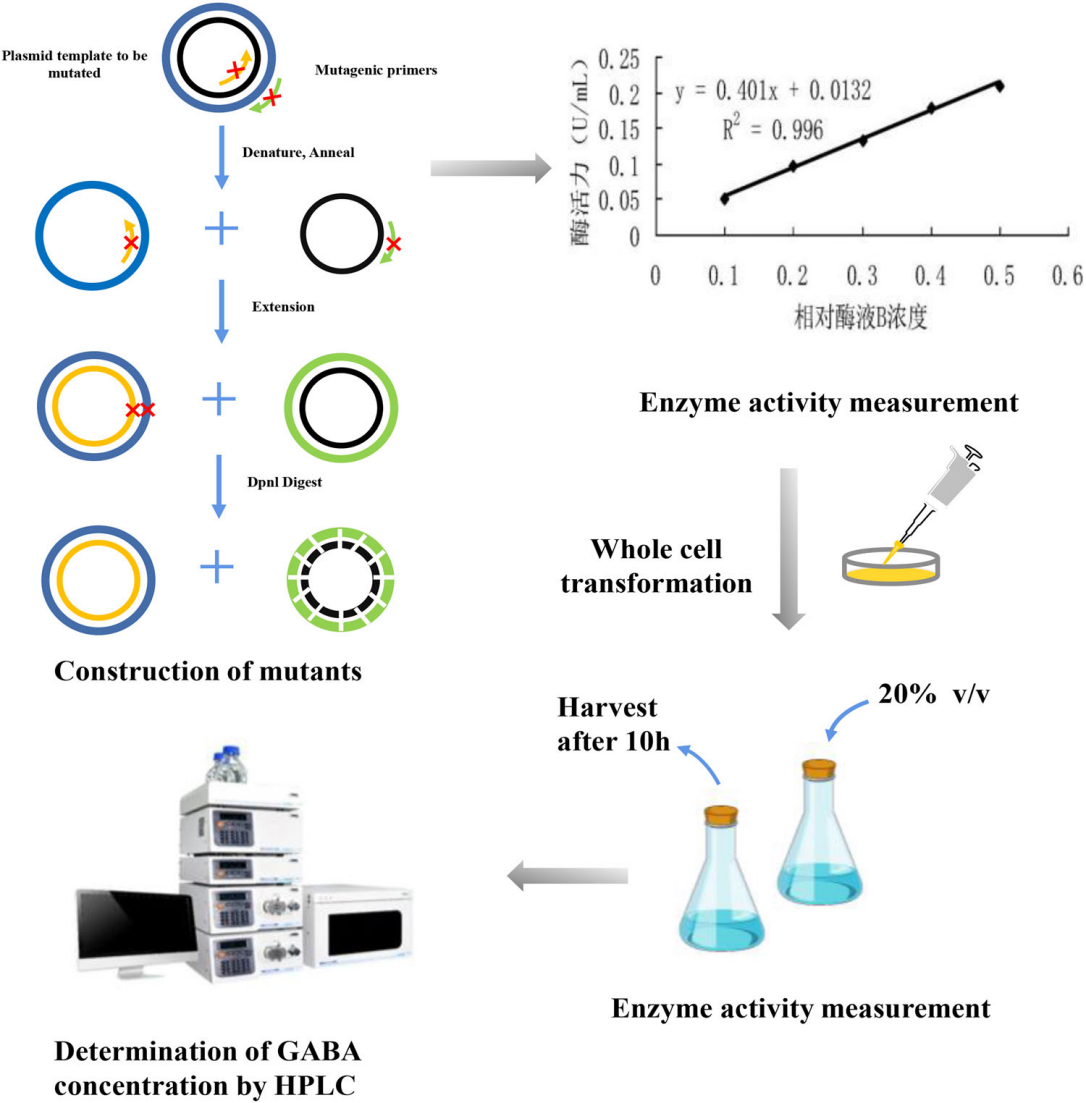
Schematic diagram of relevant experimental processes of this study.

In this study, the amino acid residues related to the catalytic performance of GAD were summarized based on a homologous comparison of Gad B (the gene that encodes the B isoform of GAD) and its catalytic–substrate interactions to synthesize GABA. Additionally, the related amino acid residues were established by mutation free energy analysis. GAD before and after mutation was expressed in *E. coli* BL21(DE3). The effect of site‐directed mutation on the GAD enzymatic properties and the GABA production of the recombinant strains were further determined (as shown in Figure 1). The results provide a context for the directional transformation of enzymes and facilitate a better application of the whole‐cell transformation approach to GABA production using recombinant strains.

## 2 MATERIALS AND METHODS

### 2.1 Experimental samples and reagents

Table 1 shows the strains, plasmids and primers (synthesized by Shanghai Shenggong) used in the present study. The restriction endonucleases *Bam* HI and *Hind* III, T4 DNA ligase, *Dpn* I digestive enzyme, Pyrobest DNA polymerase, kanamycin and nucleic acid dye were purchased from Baoriyi Biotechnology (Beijing) Co., Ltd., and LB medium from Oxoid, UK. Glucose, sucrose and NH_3_·H_2_O were purchased from Tianjin Kemio Chemical Reagent Co., Ltd.; KH_2_PO_4_ from Tianjin Damao Chemical Reagent Factory; MgSO_4_·7H_2_O from Nanjing Aoduofuni Biotechnology Co., Ltd.; and acetonitrile (LC‐grade) was purchased from Shanghai McLean Biochemical Technology Co., Ltd.

**TABLE 1:** Strains, plasmids and primers used in this study

Strain, plasmid or primer	Characteristics	Source
Strain		
*Escherichia coli* BL21(DE3)	Plasmid‐expressing host bacterium	Lab stock
*Escherichia coli* K‐12	γ‐Aminobutyric acid‐producing strain	Lab stock
*E. coli* BL21(DE3)‐Gad B	Recombinant strain capable of expressing glutamic acid decarboxylase	This study
*E. coli* BL21(DE3)‐Gad B‐T62S	Recombinant strain capable of expressing mutant glutamate decarboxylase	This study
*E. coli* BL21(DE3)‐Gad B‐Q309A	Recombinant strain capable of expressing mutant glutamate decarboxylase	This study
Plasmid		
pET‐30a(+)	Overexpression vector, kanamycin resistance	Lab stock
pET‐30a(+)‐Gad B	pET‐30a(+) carrying Gad B from *Escherichia coli* K‐12	This study
pET‐30a(+)‐Gad B‐T62S	pET‐30a(+) carrying mutated Gad B from *Escherichia coli* K‐12	This study
pET‐30a(+)‐Gad B‐Q309A	pET‐30a(+) carrying mutated Gad B from *Escherichia coli* K‐12	This study
Primer (5′→3′)		
P‐Gad B	Forward: 5′‐ACGC(GTCGAC)ATGGATAAGAAGCAAGTAACGGA‐3′ (*Bam* HI) Reverse: 5′‐CCG(CTCGAG)TCAGGTATGTTTAAAGCTGTTCTGT‐3′ (*Hind* III)	
P‐Gad B‐T62S	Forward: 5′‐GGTCTGGCAGAAAGAGGCCAGGTTCTGAC‐3′ Reverse: 5′‐GTCAGAACCTGGCCTCTTTCTGCCAGACC‐3′	
P‐Gad B‐Q309A	Forward: 5′‐GATGGCAAAAGTACCAATTGCACCACCCAGGTAGTCAACG‐3′ Reverse: 5′‐ CGTTGACTACCTGGGTGGTGCAATTGGTACTTTTGCCATC‐3′	

### 2.2 Experimental method

#### 2.2.1 Construction of mutants

The crystal structure of homo‐hexameric Gad B (pdb: 1 pmm) was downloaded from the protein data bank (http://www.rcsb.org).Conserved domains and sequence motifs were analyzed using the online software MEME (http://meme‐suite.org/), EMBI (http://www.ebi.ac.uk/Tools/msa), and WebLogo (http://weblogo.threeplusone) to select the mutation sites [22, 23, 24]. Protein structure and protein ligand interaction analyses were performed using the three‐dimensional structure visualization software VMD (http://www.ks.uiuc.edu/Research/vmd) [25]. The mutation energy (ΔΔG) after point mutation was predicted using the online software PoPMiSiC (http://soft.dezyme.com/query/create/pop) [26]. The software is based on the change value of the folding free energy (ΔΔG) of mutants to predict all possible point mutations and reasonably select mutation sites.

For the acquisition of recombinant strains, the Gad B gene(GenBank accession no. EF551356.1) derived from *E. coli* K‐12 was used as template and P‐Gad B was used as the primer for PCR amplification. The recombinant plasmid pET‐30a(+)‐Gad B was obtained by enzyme digestion and gel recovery after linking with pET‐30a(+). After correct sequencing, the amplified DNA was transformed into *E. coli* BL21(DE3) competent cells to obtain the recombinant strain *E. coli* BL21(DE3)‐Gad B. The extracted recombinant plasmid pET‐30a(+)‐Gad B was used as a template and P‐Gad B‐T62S or P‐Gad B‐Q309A was used as a primer for PCR amplification. The PCR products were added to *Dpn* I enzyme to digest the methylation template and then transformed into *E. coli* BL21(DE3) competent cells to obtain the recombinant strains *E. coli* BL21(DE3)‐Gad B‐T62S and *E. coli* BL21(DE3)‐Gad B‐Q309A.

For the purification and expression of GAD, the recombinant strains were selected and inoculated into LB liquid medium supplemented with kanamycin (final concentration 50 μg/mL) for overnight culture. The recombinant strains at 1% were then cultured in TB medium containing kanamycin (final concentration of 50 μg/mL) at 37°C with shaking (200 r/min) until the OD_600_ was 0.7–0.8, and further induced with 0.2 mM isopropyl thiogalactoside (IPTG). The induced bacterial solution was centrifuged at 12,000 r/min (4°C) for 4 min, the culture medium was discarded and the bacteria were collected. The bacteria were then suspended in PBS buffer solution (pH 7.4) and the cells in the suspension were broken by ultrasonic treatment. The bacteria were centrifuged at 12,000 r/min (4°C) for 10 min and the supernatant was filtered using a 0.22 μm membrane to obtain GAD crude enzyme solution. The GAD crude enzyme solution was purified by using the Ni‐NTA method [27, 28].

#### 2.2.2 Determination of GAD enzyme characteristics before and after mutation

The amount of enzyme required to catalyze the substrate to produce 1 μmol GABA per minute in the reaction solution is 1 activity unit (U). The Lineweaver–Burk Plot method [29] was used to investigate the kinetic parameters of GAD. To determine the enzyme activity, 980 μL of substrate buffer (including 0.05 mM pyridoxal phosphate (PLP) and 200 mM L‐glutamate sodium) was prepared, purified, and diluted to a concentration of 10 mg/mL Gad B enzyme protein solution. The total reaction solution was 1 mL after mixing, and the pH of the reaction solution was adjusted by phosphate buffer. After 30 min of reaction under different conditions, the reaction was terminated in an 80°C hot water bath for 10 min, centrifuged and the supernatant was filtered through a 0.22 μm membrane. The GABA content of the supernatant was determined using high‐performance liquid chromatography to investigate the catalytic performance of GAD before and after mutation.

#### 2.2.3 Whole cell transformation to produce GABA

*E. coli* BL21(DE3)‐Gad B, *E. coli* BL21(DE3)‐Gad B‐T62S, and *E. coli* BL21(DE3)‐Gad B‐Q309A recombinant strains stored in glycerol tubes were cultured in LB slant medium. After that, the inclined plane strains were selected and activated in the seed medium to obtain seed liquid. The seed solution was inoculated at 20% in the fermentation medium for high‐density fermentation (NH_3_·H_2_O was added to maintain pH at 7.0, temperature at 35–37°C and dissolved oxygen level at 40%–50%). When the final OD_600_ value reached about 30, 0.2 mM IPTG was added for induction. After induction, the cells were collected after centrifugation for the whole cell transformation experiment at pH 6.0 and 33–35°C, and the dissolved oxygen level was maintained at 40%–50% by flowing NH_3_·H_2_O. The seed culture medium was composed of: 2 g/L glucose, 10 g/L sucrose, 1 g/L KH_2_PO_4_, 1 g/L urea, 0.5 g/L MgSO_4_·7H_2_O, 0.3 g/L succinic acid, 1 × 10^−4^ g/L calcium pantothenate, 1 × 10^−5^ g/L vitamin B2 and 1 × 10^−5^ g/L biotin. The transformation medium was: 400 g/L L‐glutamic acid, 50 μg/mL kanamycin, and 60 μmol/L PLP; NH_3_·H_2_O was added to control the pH of transformation at about 6.0.

#### 2.2.4 Analytical method

A spectrophotometer was used to measure the OD changes of fermentation broth corresponding to each strain. An SBA‐40E biosensor was used to monitor the glucose and L‐glutamate concentrations in the fermentation and transformation processes, respectively. The high‐performance liquid phase was used to monitor the concentration of GABA before and after GAD mutation according to the method specified by Meeploy and Deewatthanawong, Wang et al. [30, 31].

## 3 RESULTS AND DISCUSSION

### 3.1 Determining the mutation point

**FIGURE 2:**
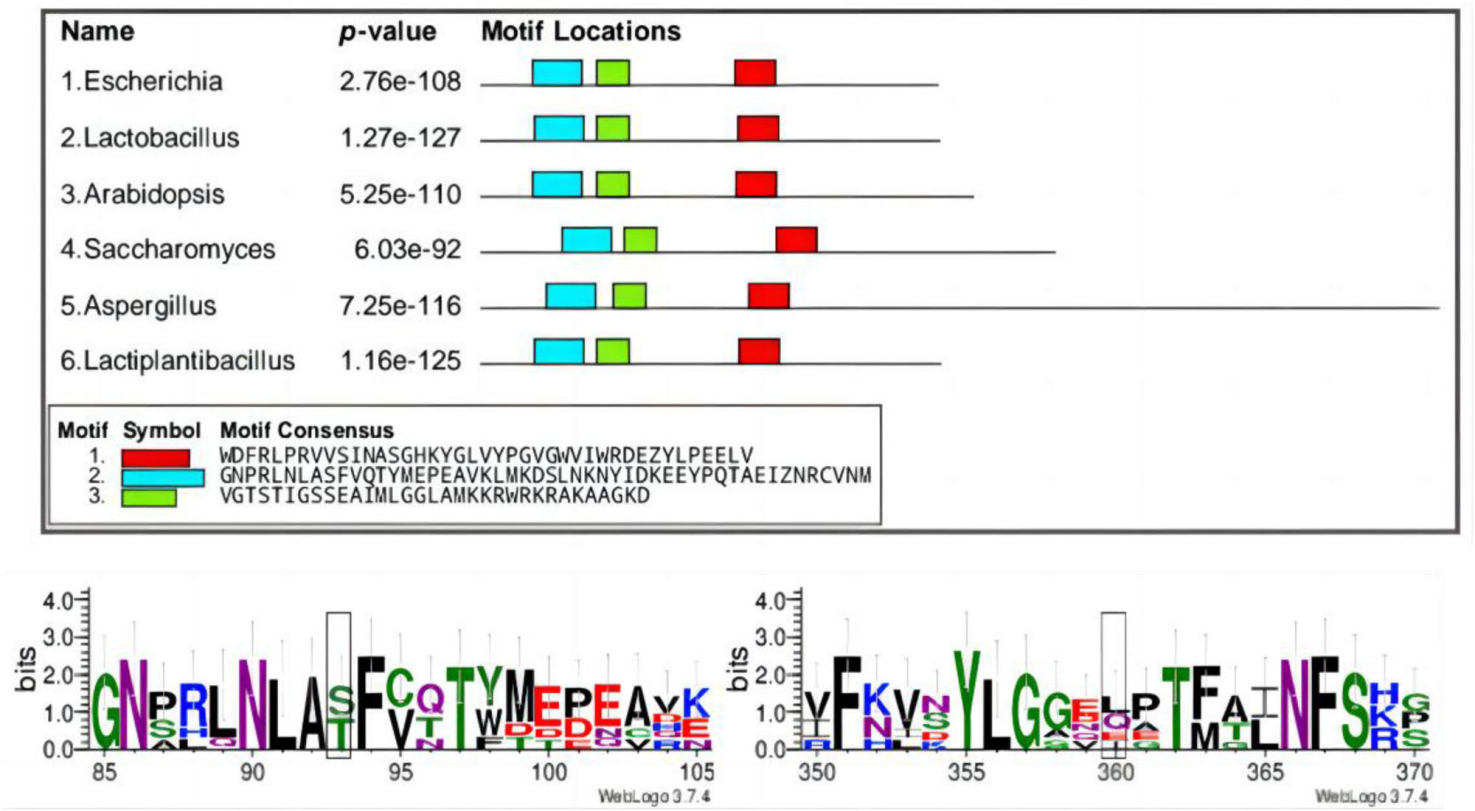
Conserved domains and sequence motifs.

The stability of an enzyme is usually the guarantee of its production and application, and the stability of structure is the basis of enzyme stability. In the process of biological evolution, the arrangement of amino acids will change with changes of the internal and external environment, but the conserved domain in this process is basically unchanged, which is closely related to structural stability [32]. Through multiple sequence alignment of several sources of GAD, it was found that they all have three conserved domain sequences (as shown in Figure 2). In Gad B site‐directed mutation, selecting non‐conserved amino acid mutations in the conserved domain structure will increase the effectiveness of the mutation and reduce the damage to the original structure when the domain is well‐defined. After multi‐sequence alignment screening, it was found that residues 62 and 309 of Gad B were located in the conserved domain, and they were not highly conserved amino acids.

**FIGURE 3:**
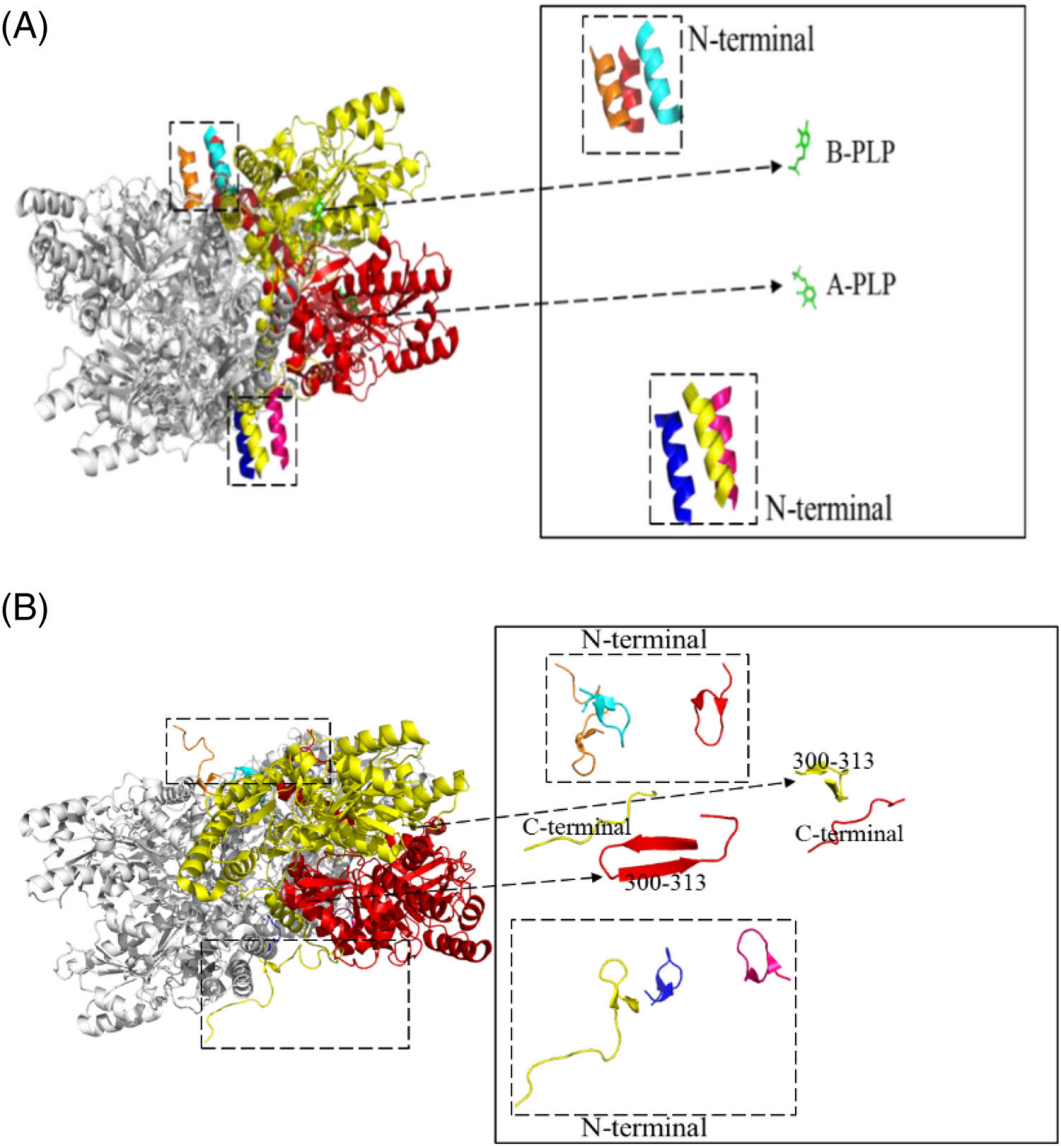
Gad B protein structure under acidic (A) and neutral (B) conditions.

The structure of Gad B under neutral and acidic conditions was analyzed and found to be a hexamer composed of six subunits. The difference was that the six subunits composing the hexamer under acidic conditions were the same (as shown in Figure 3A), while the six subunits composing the hexamer under neutral conditions were different (as shown in Figure 3B). Furthermore, Gad B has the ability to catalyze the synthesis of GABA from L‐glutamate under acidic conditions, but loses this ability under neutral conditions [33]. Structural analysis of Gad B under two pH environments showed that the C‐terminus of Gad is far away from the active catalytic center under acidic conditions, while the active center of PLP is exposed, which promotes contact between the substrate and the active site. Meanwhile, the N‐terminus of each subunit of Gad was found to be “helical” [34] and in contact with adjacent subunits, forming a steady‐state structure under acidic conditions (as shown in Figure 3A). Under neutral conditions, the C‐terminus of Gad B is close to K276 (that is, the active catalytic site binding with cofactor PLP) and forms the occupied conformation, thus losing the catalytic effect of Gad B on L‐glutamate [35]. At the same time, when the C‐terminus of Gad B extends to the active catalytic center under neutral conditions, it contacts the β‐lamellar structure formed by amino acids at positions 300–313 of the adjacent subunits, which is also a key factor affecting the catalytic effect of Gad B (as shown in Figure 3B). It can be concluded that the change of amino acid structure of Gad B under neutral conditions is the main reason for the change of its catalytic performance. In order to solve the limitation of GABA production by Gad B under neutral conditions, the site‐directed mutation method is proposed to modify the amino acid residues that interact with the active center under neutral conditions. Meanwhile, in order to verify the effect of site‐directed mutation on the catalytic performance of the enzyme and reveal the cause of the change, the relevant amino acid residues in the β‐lamellar structure of residues 301–313 react with the C‐terminus extending to the active center under neutral conditions.

**FIGURE 4:**
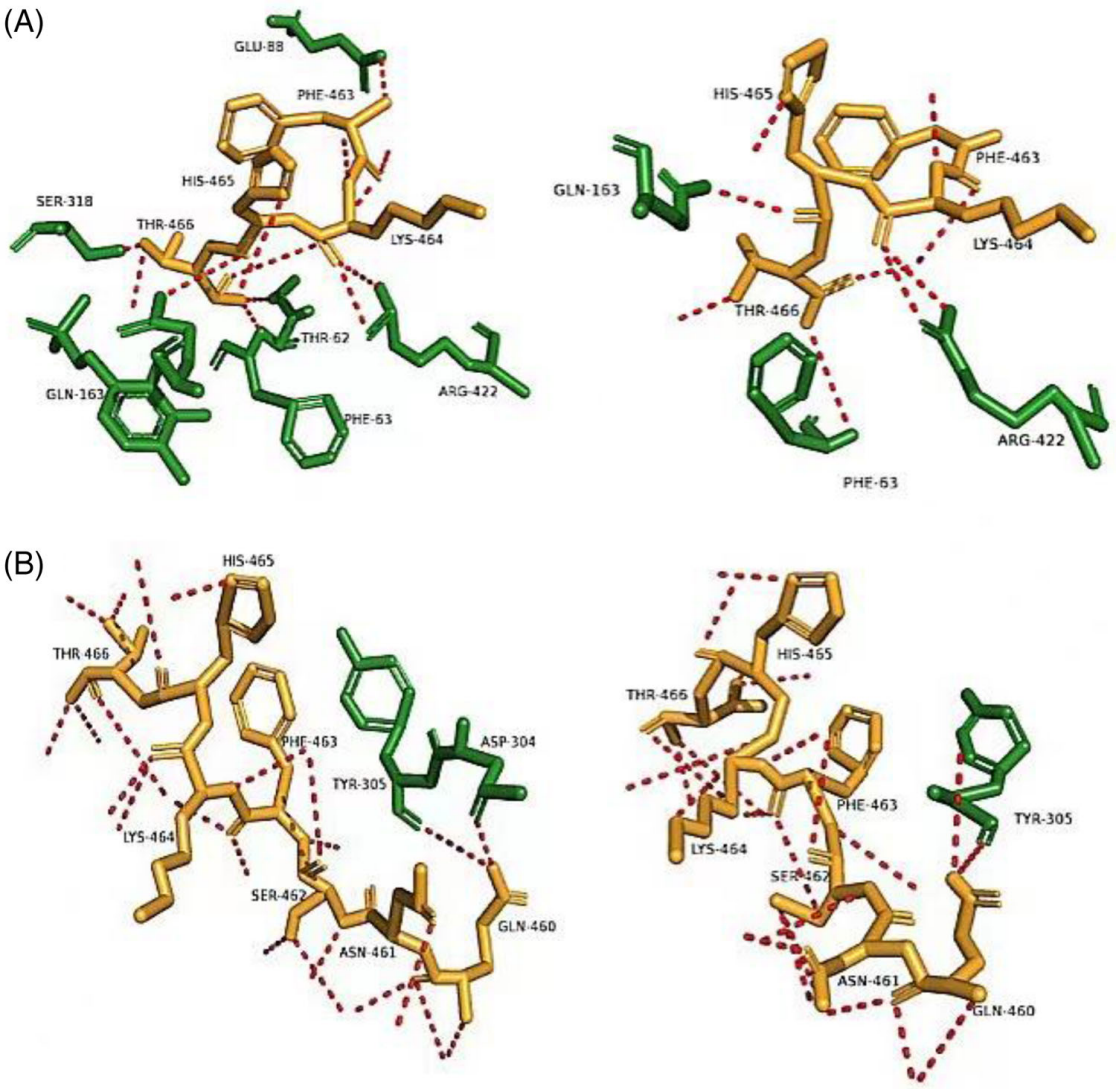
Conformation of Gad B under neutral conditions before and after mutation: amino acid residues at position 62 (A) and position 309 (B).

Submit the three‐dimensional structure of GAD enzyme to online software PoPMiSiC, predict the change of folding free energy(ΔΔG) of each mutant amino acid of GAD enzyme, and select the amino acid residue with negative ΔΔG and significant change as the mutation site. Based on the conserved domain analysis and multi‐sequence alignment analysis, and on the change of mutation energy (ΔΔG < 0), it was determined that the 62nd amino acid residue of Gad B was mutated from threonine to serine. After mutation, the amino acid residues at position 466 lost their interaction with the amino acid residues at position 62 (as shown in Figure 4A), which weakened the occupation of the C‐terminus at the active catalytic center of PLP and changed the catalytic effect of Gad B near the center pH. In addition, at neutral pH, the β‐lamina formed by amino acid residues 300–313 interacts with the C‐terminus to form an envelope conformation of the active catalytic center of PLP, causing the neutral of the exposed active catalytic center of PLP to shrink significantly into the interior of Gad B. It is difficult for the substrate L‐glutamate to enter the active pocket, which weakens its catalytic performance. Position 309 at the end of the β‐lamellar formed by the amino group of Gad B's amino acids 300–313 is the hydrophilic amino acid glutamine, which has a flexible conformation and is often used as a binding site for various proteins. Therefore, it was decided to mutate Gad B into a hydrophobic amino acid (for proteins, large, deep hydrophobic cavities are critical for substrate binding to the receptor), in an attempt to weaken the interaction between Gad 309 as a hydrophilic amino acid and the C‐terminus while expanding the channel of the substrate into the catalytic pocket. Such a mutation changes the structure and orientation of Gad B under neutral conditions [36, 37], and the contact effect is weakened (as shown in Figure 4B). Second, the R group of the amino acid residues that form the β‐lamellar must not be too large, so stretching is conducive to the chain, under the condition of the peptide chain extension, most amino acids hydrophilic interaction with the surrounding water, a large number of hydrophilic amino acid residues of side chain groups and melting by solvent, which contribute to the formation of hydrophobic internal role, hydrophobic packaging to form active center cavities is also a major determinant of protein stability [38]. Guo et al. [39], using online prediction software PoPMiSiC, to predict the change of free energy of single point mutation of *Bacillus subtilis* chitosanase Bs Csn46A, the specific activity of the three mutants increased 1.69, 1.97, and 2.15 times, respectively. Similarly, Zhu et al. [40], screened the mutant aromatic sulfatase K253H with improved thermal stability by using PoPMiSiC. These studies show that the online prediction software PoPMiSiC is used to calculate the change of the ΔΔG of each amino acid site in the protein sequence to help design the site‐directed mutation of the enzyme, which may change the interaction between the amino acid residues in the local region of the enzyme molecule and predict the change of the enzyme stability after the amino acid point mutation.

### 3.2 Construction and verification of mutants

**FIGURE 5:**
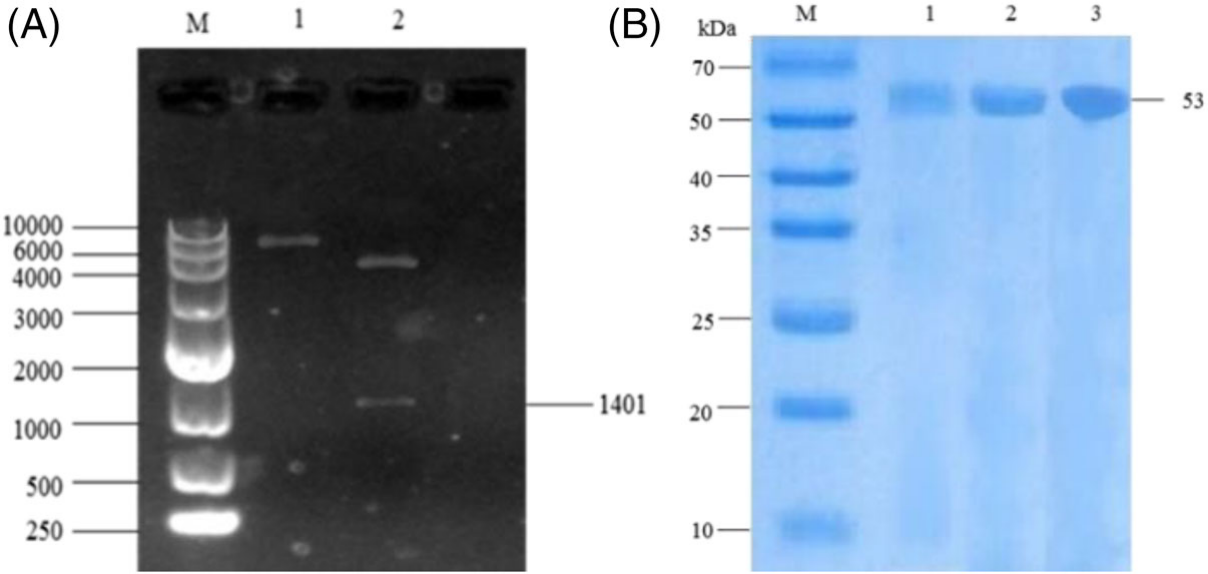
（A） Double enzyme digestion verification map (M, marker; 1, pET‐30a(+)‐Gad B recombinant plasmid band; 2, double enzyme digestion verification band); (B) GAD polyacrylamide gel electrophoresis map (M, marker; 1, Gad B protein band; 2, Gad B‐T62S protein band; 3, Gad B‐Q309A protein band).

After the mutation site was determined, the DNA of *E. coli* K‐12 was extracted as the amplification template, and the 1401 bp target fragment of *gad B* was amplified with the primer P‐Gad B. The amplified target fragment was linked to plasmid pET‐30a(+) for double‐digestion verification. The recombinant plasmid pET‐30a(+)‐Gad B was successfully obtained (as shown in Figure 5a). The plasmid containing the mutant site was further amplified by PCR with primer P‐Gad B‐T62S; the amino acid residue 62 of Gad B was mutated from tryptophan to serine, and residue 309 was mutated from glutamine to alanine. The plasmid obtained above was transformed into *E. coli* BL21(DE3) cells to obtain the recombinant strains *E. coli* BL21(DE3)‐Gad B, *E. coli* BL21(DE3)‐Gad B‐T62S, and *E. coli* BL21(DE3)‐Gad B‐Q309A. The induced expression and SDS‐PAGE analysis of the strains before and after mutation were further carried out. The expression vector pET‐30a(+) contained a His protein coding sequence. The crude enzyme solution of GAD was purified by Ni‐NTA and verified by SDS‐PAGE. The protein band appeared at about 53 kDa (as shown in Figure 5B). Thus, this indicated the successful intracellular expression of GadB in recombinant strains *E. coli*.

In theory, three codon saturation mutagenesis will give rise to a library with 8000 members if each mutation is represented equally. Mutations are bidirectional. Some may increase their thermal stability, while others may reduce their thermal stability. In this study, some mutants with residual activity higher than wild type were collected and sequenced through repeated screening. Because some mutants had the same amino acids sequence, we actually only obtained two thermal stable mutants. The purpose of our study was to find and characterize thermal stable *E.coli* GadB mutants. We only studied this two mutants in detail and found Gad B‐Q309A was the most stable one among them.

### 3.3 Verification of enzymatic properties

**FIGURE 6:**
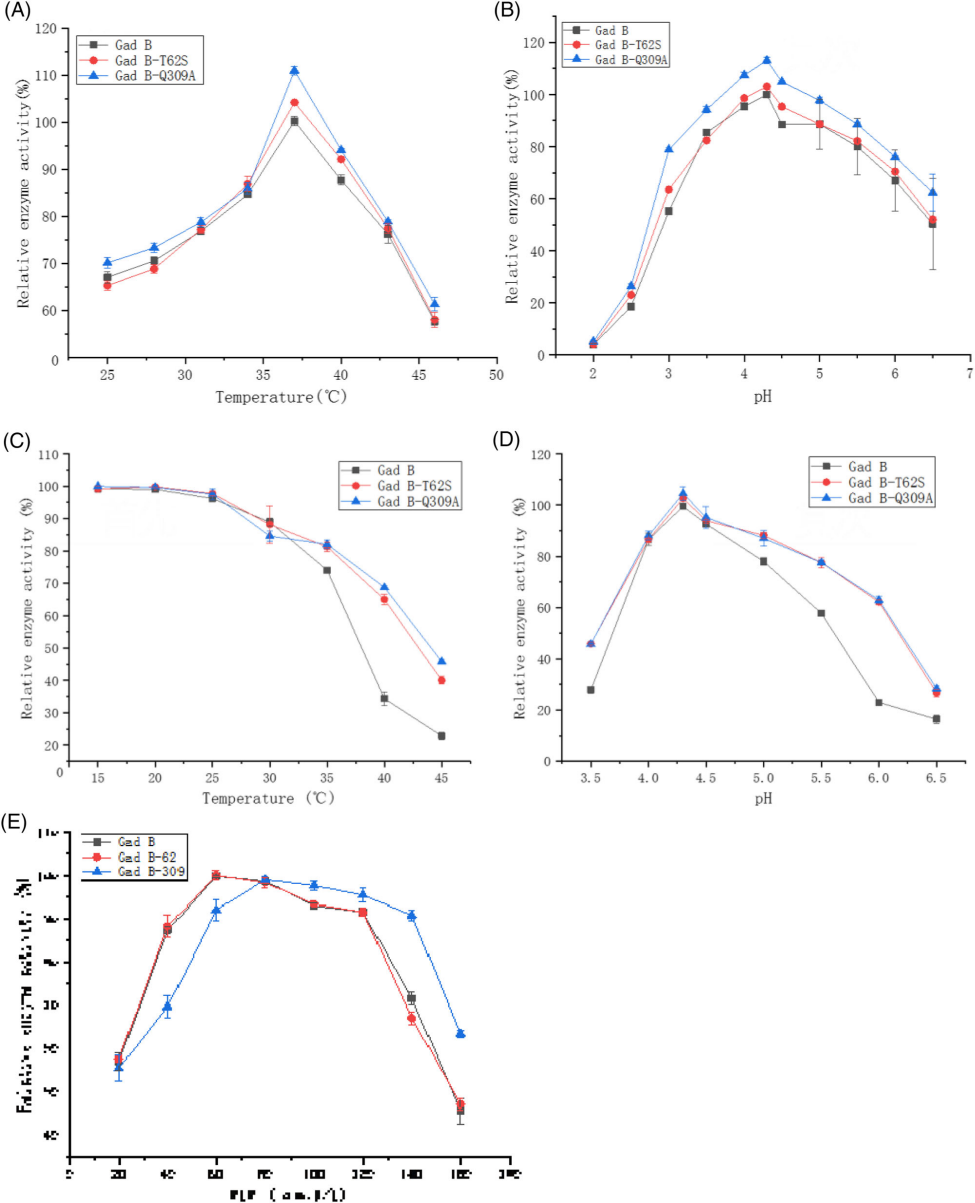
Effect of different conditions on enzyme activity. (A)optimum temperature determination diagram; (B) optimal pH determination diagram; (C) temperature stability; (D) pH stability; (E) optimal PLP concentration diagram.

Temperature and pH characteristics: at pH 4.5, the optimum temperature of the enzyme was determined (as shown in Figure 6A). Gad B, Gad B‐T62S, and Gad B‐Q309A showed enzyme activity in the range of 25 to 46°C, the highest activity being found at 37°C. The activity of Gad B‐T62S and Gad B‐Q309A was higher than that of Gad B at 37°C. At 37°C, the optimal pH of the enzyme was determined (as shown in Figure 6B). Gad B, Gad B‐T62S, and Gad B‐Q309A showed enzyme activity in the range of pH 2.0 to 6.5, the activity being highest at pH 4.3, and the activity of Gad B‐T62S and Gad B‐Q309A was higher than that of Gad B at pH 4.3. At 37°C and pH 4.3, both Gad B‐T62S and Gad B‐Q309A mutants showed increased enzyme activity compared with the wild‐type Gad B. This preliminarily determined the optimum reaction conditions for GAD and provided corresponding parameters for the verification of GAD enzyme stability.

The enzyme was stored at 15 or 20°C for 12 h, and then used for validation of enzyme activity. Due to low temperature inhibition, its activity was not affected. Gad B was stored at 25, 30, and 35°C for 12 h, and then used to verify the enzyme activity. The enzyme activity was damaged when the temperature increased, and the enzyme stability decreased sharply when the temperature exceeded 35°C; under the same conditions, Gad B‐T62S and Gad B‐Q309A were less affected. At 45°C and pH 4.3, the thermal stability of Gad B‐T62S and Gad B‐Q309A mutants compared with wild‐type Gad B increased from 22% to 41% and 49%, respectively (as shown in Figure 6C), indicating that the thermal stability of the enzyme is improved to a certain extent at higher temperatures. At 20°C, the enzyme was stored in buffer with different pH for 12 h and then reacted in buffer with substrate. The mutant strain maintained more than 60% of the relative enzyme activity in the range of pH 4.0–6.0, while the stability of wild‐type Gad B was relatively poor. At 37°C and pH 6.5, the pH stability of Gad B‐62 and Gad B‐309 mutants compared with the wild‐type Gad B increased from 18% to 24% and 28%, respectively (as shown in Figure 6D), indicating that the stability of the enzyme is improved to a certain extent when it is close to neutral pH.

Effects of PLP supplemental level on enzyme properties before and after mutation: the enzyme activity of wild‐type Gad B and Gad B‐T62S mutants was highest when using the coenzyme PLP at a concentration of 60 μmol/L. With an increase of PLP concentration, the catalytic capacity of Gad B and Gad B‐T62S was not significantly promoted, and when the PLP concentration reached 160 μmol/L, PLP significantly inhibited the enzyme activity of Gad B and Gad B‐T62S. The mutant Gad B‐Q309A did not have optimal enzyme activity at 60 μmol/L PLP. However, at 80 μmol/L PLP, the enzyme activity of Gad B‐Q309A was significantly increased, which suggests that the optimal PLP concentration for Gad B‐309 is 80 μmol/L (as shown in Figure 6E); when the PLP concentration was 160 μmol/L, PLP could also inhibit Gad B‐309, but the enzyme activity of Gad B‐309 was higher than that of Gad B and Gad B‐T62S. In the later production of GABA, this experiment provides a reference for PLP concentration.

Kinetic parameters K_m_ and V_max_: it can be seen from Table 2 that the K_m_ value of GAD after mutation is significantly smaller than that before mutation, and the V_max_ of GAD after mutation is significantly higher than that before mutation; according to the K_m_ value, the V_max_ of Gad B‐62 and Gad B‐309 is 2.37 and 2.71 times higher than that of Gad B, respectively. In addition, according to the K_m_ value, the affinity between GAD and substrate was also enhanced after mutation, achieving the desired result of site‐directed mutation.

**TABLE 2:** Kinetic parameters of enzymes before and after mutation

Enzyme	K_m_ (mM)	V_max_ (U/mg)
Gad B	11.3 ± 2.1	32.1 ± 2.4
Gad B‐T62S	7.3 ± 2.5	76.1 ± 3.1
Gad B‐Q309A	7.2 ± 3.8	87.3 ± 1.1

### 3.4 Whole cell transformation produces GABA

**FIGURE 7:**
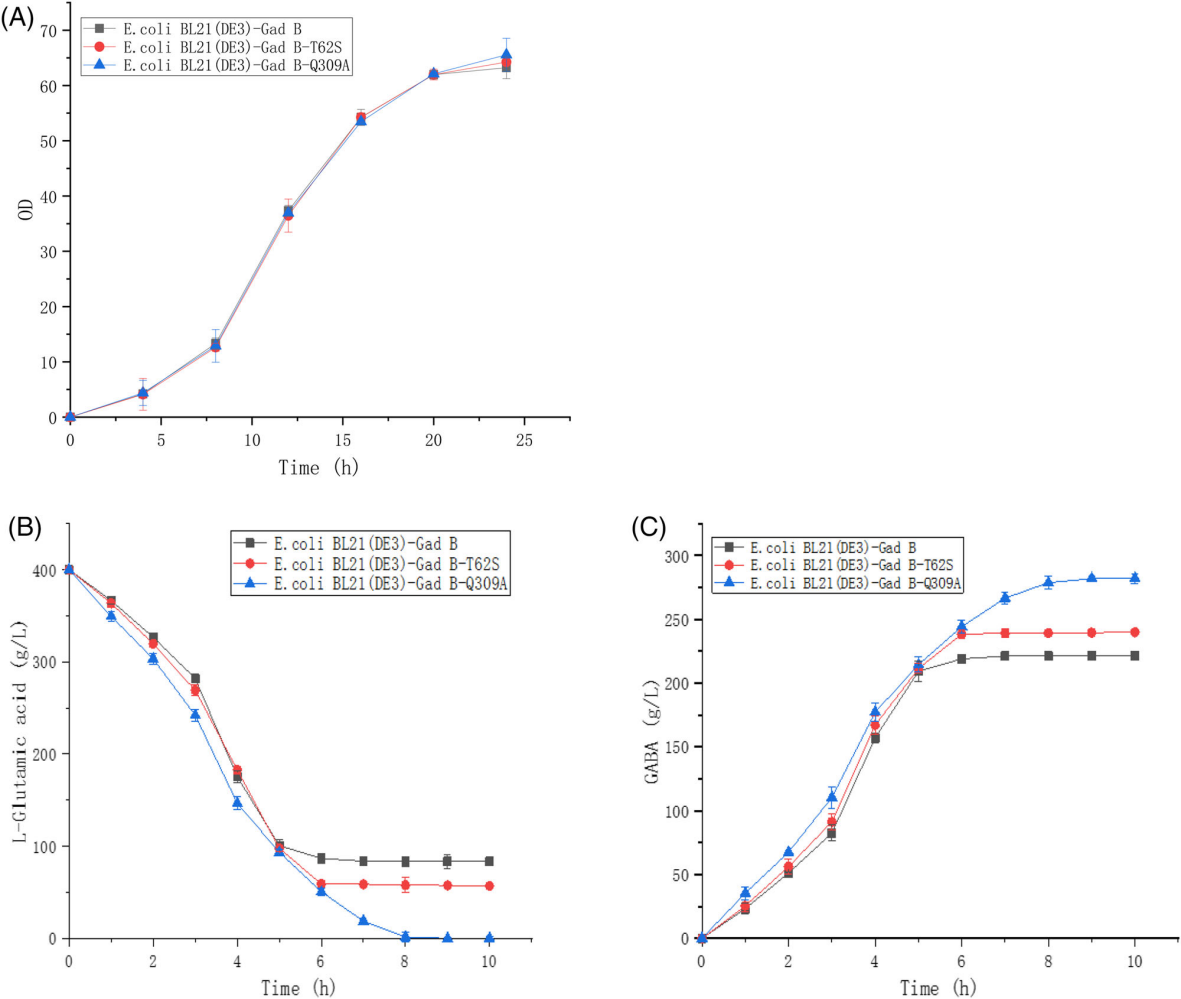
Whole‐cell conversion of L‐Glutamate into γ‐aminobutyric by wild‐type and mutant type (A) Changes of OD value in fermentation tank over time; (B) L‐glutamate consumption curve in transformation; (C) GABA generation curve in transformation.

After seed solution was inoculated in a 5 L fermentation tank, the three strains entered logarithmic growth phase at about 8 h and left logarithmic growth phase at about 12 h, and the OD_600_ value of the three recombinant strains at this stage was around 30 (as shown in Figure 7A). At this time, IPTG was added to the fermentation broth and the concentration was controlled to 0.2 mM. The fermentation temperature was controlled at 35–37°C and the dissolved oxygen was maintained at 40%–50%. In the later stage of fermentation, attention should be paid to the sterile air flux and stirring speed in time.

At this point, the bacteria were collected by centrifugation and suspended with an appropriate amount of sterilized ultra‐pure water to enter the whole cell transformation experiment. The cycle of whole cell transformation and production of GABA was 12 h, and 400 g/L L‐glutamate substrate was added. The substrate L‐glutamate is consumed when it is put in (as shown in Figure 7B). Meanwhile, the bacteria have the highest utilization efficiency of L‐glutamate at 3–5 h. In the 6th hour of transformation, the concentration of L‐glutamate decreases slowly, which may be the reason for the decrease of bacterial biological activity. The concentration of L‐glutamate did not change at all. Since the whole cell transformation lasted for about 6 h, L‐glutamate consumption of the three strains did not change, so termination of the whole cell transformation experiment could be considered after 5–6 h. In this experiment, after measuring the residual concentration of L‐glutamate in the fermentation broth after 8 h of transformation, the concentration of L‐glutamate in the whole cell transformation broth corresponding to the three recombinant strains *E. coli* BL21(DE3)‐Gad B, *E. coli* BL21(DE3)‐Gad B‐T62S, and *E. coli* BL21(DE3)‐Gad B‐Q309A was 83.43, 56.92, and 1.03 g/L, respectively. Strains containing GAD before and after mutation can effectively utilize the substrate L‐glutamate for the whole cell transformation experiment, and the degree of L‐glutamate utilization by *E. coli* BL21(DE3)‐Gad B‐T62S and *E. coli* BL21(DE3)‐Gad B‐Q309A is higher than that by *E. coli* BL21(DE3)‐Gad B, proving that the mutant strains have the potential to produce GABA, and the forward mutant strains were successfully obtained.

The whole cell transformation selected can be said to be between fermentation and enzyme transformation. Compared with the consumption of L‐glutamic acid in direct fermentation, whole cell transformation may be due to the directness and specificity of GAD enzyme. As soon as it enters the whole cell transformation experiment, L‐glutamic acid is consumed in large quantities. Compared with enzyme‐catalyzed GABA production, whole cell transformation retains the integrity of the cell and maintains a cascade between various enzymes in the cell [41]; further, the whole cell‐transformed bacteria can be reused, such as in continuous fermentation or transformation. According to the L‐glutamate consumption curve, the consumption rate of L‐glutamate substrate by strains *E. coli* BL21(DE3)‐Gad B‐T62S and *E. coli* BL21(DE3)‐Gad B‐Q309A was significantly higher than that by strain *E. coli* BL21(DE3)‐Gad B. With the passage of time, the influence of environment on enzyme stability limits the ability of strain *E. coli* BL21 (DE3)‐Gad B to produce GABA, while strains *E. coli* BL21 (DE3)‐Gad B‐T62S and *E. coli* BL21 (DE3)‐Gad B‐Q309A can stably consume L‐glutamate in the middle and late stages of transformation compared with strain *E. coli* BL21 (DE3)‐Gad B, and the effect of *E. coli* BL21 (DE3)‐Gad B‐Q309A is better. This further shows that site‐directed mutation can effectively improve the stability of GAD and can be used in the production of GABA.

According to the GABA formation curve (as shown in Figure 7C), the accumulation of GABA increased significantly at 1–4 h, the synthesis rate of GABA slowed down at 5 h and there was almost no increase at 7 h. *E. coli* BL21(DE3)‐Gad B‐T62S and *E. coli* BL21(DE3)‐Gad B‐Q309A had a higher catalytic rate of GABA synthesis than *E. coli* BL21(DE3)‐Gad B. After one cycle of whole cell transformation, the concentration of GABA in the corresponding fermentation broth of recombinant strains *E. coli* BL21(DE3)‐Gad B, *E. coli* BL21(DE3)‐Gad B‐T62S, and *E. coli* BL21(DE3)‐Gad B‐Q309A was 219.09, 238.42, and 276.66 g/L, respectively, and the transformation rates were 78.02%, 85.04%, and 98.58%, respectively. The results show that GAD from wild *E. coli* was expressed in competent *E. coli* BL21(DE3) cells, which effectively transformed L‐glutamate to GABA, significantly shortened the production cycle and solved the problem of weakening enzyme protein activity due to long‐term fermentation.

In whole cell transformation, GABA is mainly obtained by the enzymatic method. In order to ensure sufficient biological activity of cells, various nutrients in the culture medium should be strictly controlled during early fermentation, and an appropriate IPTG concentration should be selected for induction at the appropriate time. In the experiment, whole cell‐transformed bacteria are generally collected by centrifugation. The centrifugation process may cause damage to the bacteria. We can try to collect the bacteria by natural sedimentation in order to provide them a certain degree of protection. In this experiment, in the later stage of whole cell transformation, due to the lack of increase of bacterial activity and the accumulation of products in the fermentation tank, the experiment was terminated. The GABA production efficiency of this process is high, but a large number of bacteria are discarded after being used in a short time. In a later experiment, we can try to recover the bacteria for whole cell transformation to produce GABA while achieving continuous transformation to produce GABA so that the bacteria can be reused. Compared with direct feeding, continuous feeding may alleviate the problem that the concentration of L‐glutamate in the fermentation broth is too high and affects the performance of bacteria. Of course, too high a concentration of GABA will also inhibit the rate of GABA production, so that the GABA produced in cells cannot be transported out of cells in time, thus affecting the yield of GABA. Therefore, it is necessary to effectively isolate the GABA produced during whole cell transformation, which should be paid attention to in future research.

At present, the yield of GABA produced by *E. coli* or *Lactobacillus brevis* is higher than that by other strains (such as *Corynebacterium glutamicum*, yeast, *Aspergillus oryzae*, etc.) [42]. Plokhov et al. [43]. reported the expression of *E. coli* K‐12 GAD in *E. coli* BL21(DE3), and the whole cell was transformed into GABA with a transformation rate of 98.6%. Zhang et al. [44]. Screened a strain of *L. brevis* GLB‐127 and transformed it into GABA in a 10 L fermentation tank, with a conversion rate of 98.5%, which is a high level of GABA production at present. Although *C. glutamicum* itself does not have a GABA metabolic system, it is a strain that produces a high level of the GABA precursor L‐glutamate. The method of recombining the GAD system into *C. glutamicum* and producing GABA with a recombinant strain has also attracted attention, which also effectively utilizes the L‐glutamic acid produced in its own metabolic system [45]. In this study, the GABA conversion rate of the recombinant strain *E. coli* BL21 (DE3)‐Gad B‐Q309A was the highest, 98.58%, but did not reach the level obtained in relevant studies. However, this experiment only verified the GABA production ability of the recombinant strain, and did not refine and adjust the operating conditions of the fermentation and transformation processes. Therefore, it is necessary to optimize the fermentation and transformation conditions in the later stage to improve the GABA yield. In recent years, GABA has tended to be produced at the food and drug level. Attention should be paid to the production of GABA with GAD from probiotics [46]; however, when wild GAD is used for continuous transformation or long‐term preservation, its vitality is often damaged due to its insufficient stability, affecting the use effect [47]. Considering the strong operability and clear genetic background of *E. coli*, this experiment carried out site mutation on GAD from *E. coli* K‐12. While improving the stability of GAD, the whole cell transformation of recombinant strain *E. coli* BL21(DE3)‐Gad B‐Q309A produced GABA, and the yield reached 98.58%. This provides a certain convenience for the production of GABA and the preservation of GAD. In the following research, while making full use of the directed transformation technology of enzymes to transform GAD and improve the yield of GABA, we should also expand the transformation of other enzymes, to strengthen the application of various enzymes and facilitate the further expansion of production.

## 4 CONCLUSIONS

In this study, the Gad B gene of *E. coli* K‐12 was used as the template. The two amino acid residues affecting the catalytic performance of GAD were identified using bioinformatics methods, and the GAD before and after mutation was recombined into *E. coli* BL21(DE3). The mutated GAD enzyme was used to increase GABA production through the whole cell transformation approach. Two effective mutants, Gad B‐T62S and Gad B‐Q309A, were obtained. The enzyme activity of the mutants at different temperatures and pH was 4%–13% higher than that of the wild type. The pH stability was 6%–10% higher than that of the wild type and the temperature stability was 19%–27% higher than that of the wild type, while the catalytic activity and affinity with the substrate were found to be improved. The transformation rate of *E. coli* BL21(DE3)‐Gad B‐T62S and *E. coli* BL21(DE3)‐Gad B‐Q309A was higher than that of *E. coli* BL21(DE3)‐Gad B; the highest transformation rate was obtained for *E. coli* BL21(DE3)‐Gad B‐Q309A, reaching 98.58% in the present study. Overall, this study not only contributes a strategy for the directional transformation of enzymes to improve GABA production, but also enhances current knowledge related to the whole cell transformation method.

## CONFLICT OF INTEREST STATEMENT

The authors have declared no conflicts of interest.
